# Essays and Heads of Lectures on Anatomy, Physiology, Pathology, and Surgery

**Published:** 1841-04-01

**Authors:** 


ESSAYS AND HEADS OF LECTURES ON ANATOMY, PHYSIOLOGY, PATHOLOGY, AND SURGERY. By the late *Alexander Monro*, Secundus, M.D. F.R.S.E. &c., upwards of Fifty Years Professor of Anatomy and Surgery in the University of Edinburgh. With a Memoir of his Life; and copious Notes explanatory of Modern Anatomy, Physiology, Pathology, and Practice. By his Son and Successor. Illustrated by Engravings. Octavo, pp. 300. Whittaker & Co. London; Maclachlan and Stewart, Edinburgh; Fannin and Co. Dublin.

THIS is an able, agreeable, and useful volume; and cannot fail, we think, to experience a welcome reception from the profession. To the surviving pupils of the late Dr. Monro it will prove peculiarly acceptable; and will recall many interesting reminiscences of that illustrious teacher. Trained from his very boyhood to the important duties of the anatomical chair, under the eye of his father—the first Monro—who may justly be considered as the founder of the Edinburgh Medical School, and who, by his pre-eminent talents and reputation, was so well calculated to kindle both enthusiasm and emulation in the breast of his son, it is not to be wondered at that Dr. Monro, Secundus, should have displayed, at a very early age, what was looked upon as a natural aptitude for anatomical science. His long and brilliant subsequent career, and the indefatigable assiduity with which he cultivated anatomy, physiology, and pathology, for more than half a century, together with the important discoveries he achieved in these departments, have earned for him an imperishable reputation. It is undoubtedly true, and cannot be too extensively known, that Dr. Monro, Secundus, broached more of the physiology and pathology of his successors than they have been disposed to acknowledge, or have had the candour to trace to the parent source. His lectures were a mine of public wealth, from which materials were borrowed by wholesale, and produced from time to time, under a new garb, by writers, who contrived to occupy a large space in the public eye, and thus to reap a harvest of fame not their own. It would be invidious to pursue or illustrate this particular topic any farther. We shall merely content ourselves with saying, that it was high time for Dr. Monro—the present distinguished professor of anatomy in the University of Edinburgh—to come forward and vindicate, by the work before us, the just claims of his celebrated father. The task which he has thus undertaken, and which he has performed in a manner so creditable to himself, had become not only an act of filial duty, but an effort called for by honour and justice. He has been actuated by what Justinian so forcibly styles the "Jus constans, et perpetua voluntas, *suum cuique tribuendi,*" and has not only given, in the discharge of this duty, a most pleasing memorial of his eminent father, but has contributed an interesting and valuable volume to modern medical literature.

By a fortunate coincidence, Dr. Monro has been enabled to place before his readers an account, by most competent judges, of the very *first* course of lectures which his father delivered, in the year 1758; and also of his very *last* course, in the College-session of 1806-7. The former is from the pen of the celebrated Dr. Carmichael Smyth; and it is so interesting that we shall subjoin it at full length. The latter is supplied by Dr. Robertson of Northampton; to whom, by-the-bye, the volume is dedicated.

*Letter from Dr. Carmichael Smyth to Dr. Monro, Tertius.*

"Charlton, November 19th, 1817.

"Dear Sir,

I should be highly gratified could I imagine, that any observations of mine on the late Dr. Monro's career as a professor of anatomy, could contribute in the smallest degree to illustrate his character. But when I attended his lectures, I was too young, and too little acquainted with the subject, to judge or appreciate his merit. As it is, however, possible that, after a period of nearly sixty years, I may be the only one now alive who was present at his debut, I will, with much pleasure, state to you the impressions thus made upon me at the time, and which the lapse of half a century has not yet effaced. Dr. Monro, Primus, who had long filled the anatomical chair, with reputation to himself and advantage to his country, began the course of lectures in the autumn of the year 1758, and after delivering the history of anatomy, and some introductory lectures on the blood, and other general subjects, resigned his situation to his son; who, it must be acknowledged, from his father's great popularity as a public teacher, had an arduous duty to perform. He very soon convinced the public that he was equal to the task, and the students were far from regretting the change that had taken place.

Dr. Monro, Primus, had embraced the doctrine of Leeuwenhoeck respecting the blood, and taught it to the last. Your father's first lectures were employed in giving a complete refutation of this system, which was placed on the shelf for ever. He clearly proved, that the different parts of the blood were permanently and essentially distinct from each other, and entirely independent of any aggregation or combination of globules. The novelty of a doctrine of so much importance in all physiological and pathological reasoning, with the clear and luminous manner in which it was explained, operated like an electric shock on the audience, and gained him a degree of confidence, which I believe no young man ever had at starting, but which his talents were well calculated to support. The students perceived in the other parts of the anatomical course, the same clearness of demonstration, acuteness of dissertation, and accuracy of reasoning, that they admired in his refutation of Leeuwenhoeck's system. They could not help observing, that he was complete master of his subject; but that he possessed in an eminent degree another talent no less necessary for a public teacher, the proper mode of communicating his own knowledge to others. Your father enjoyed likewise, when he entered upon his public duty as a professor of anatomy, a great advantage over all his predecessors, from the high improvements made at the time in anatomical preparations, particularly from the art of injecting the bloodvessels, and corroding the parts thus injected; by means of which, he was enabled to elucidate more completely than could formerly be done, their numerous ramifications and communications.

Your father enjoyed also a pre-eminence over most other teachers of anatomy, from the use he made of mathematical calculations or diagrams, to illustrate the effect of compound muscular action. He applied this particularly to the action of the intercostal muscles, shewing the advantage arising from their oblique course. His reasoning on this subject, with a diagram which I copied at the time, I have still in my possession. But the prominent feature of your father's anatomical course, and where he shone with no borrowed lustre, was his preparations and demonstrations of the lymphatic vessels. Whether he, or his celebrated cotemporary, Dr. William Hunter of London, is entitled to the praise of their first discovery, is a point still, I believe, undecided; and I am very far from having the presumption 'tantas componere lites.' But whatever difference of opinion there may have been on this subject, there can be but one opinion respecting the high merits of both claims, and the just praise to which they are entitled, for the zeal and success with which the subject was prosecuted and illustrated by both of them.

The above observations, you will perceive, are entirely confined to the time I attended your father's lectures, which was from the Autumn 1758, for the four or five following years. His subsequent discoveries respecting the communication between the lateral ventricles of the brain—bursæ mucosæ, &c.—I leave to you and other professional gentlemen to explain and appreciate. But before I quit this subject, I have one more remark to make.

Your grandfather laid the true foundation of anatomy, and of his fame, in his accurate description of the bones; upon which your father has erected a trophy that must carry his name to the remotest ages, so long as the science of anatomy is cultivated among men.

Your's,

J. CARMICHAEL SMYTH."

(Pp. xiii.—xvi. Memoir.)

Dr. Monro, Secundus, lived to an advanced age, and continued the active duties, not only of his profession, but of the anatomical chair, till his seventy-fifth year. The infirmities inseparable from old age then coming upon him, the remainder of his honourable and useful life was necessarily passed in peaceful retirement. When he had reached his eightieth year, he was wont to become very drowsy after dinner. He also became subject to occasional headache and slight bleeding at the nose. These symptoms were the preludes to an attack of apoplexy; from which, by the unceasing attention of his friends, Dr. Rutherford and Mr. Bryce, he partially recovered. But the malady was not eradicated; his weakness gradually increased; and, after the lapse of four years, he expired, without suffering, on the 2nd of October, 1817, in the 85th year of his age.

Of his private virtues, which were on a par with his public talents, it is pleasing to speak. He was a man not only most indulgent and affectionate in his family circle; but remarkable for general and active benevolence. He was ever ready to assist the poor with his purse and professional skill; he was a subscriber to all the charitable institutions; and took a conspicuous part in the management of the Royal Infirmary. We feel it to be a duty, no less than a pleasure, to record these traits of this great and good character, inasmuch as—

> " The evil that men do lives after them ;
> The good is oft interred with their bones :"

and we are willing, as Journalists, to give historic permanency, as far as we can, to the virtues as well as the talents, for which Dr. Monro was conspicuous.

On the subject of his professional qualifications and demeanour, we shall give the testimony of a highly distinguished eye-witness—the late Dr. Gregory, of Edinburgh. We make no apology for the length of the extract; because it is a gem in its way, and is full of important instruction, especially to the junior portion of our readers. The portrait thus drawn by Dr. Gregory, is sketched with the hand of a master, and exhibits all the facility and power of that gifted individual.

" The late Dr. Monro, of Edinburgh, long, and most deservedly, enjoyed the highest eminence which any man of the medical profession ever attained in Scotland. As an able, active, and meritorious professor of anatomy and surgery, he was, for more than half a century, at the head of the great medical school of Edinburgh, and, for the greater part of that time, as a practical physician, he was unquestionably at the head of his profession in Edinburgh, and in Scotland; to many, even very distant parts of which he was often called, and from every part of which, as well as of England and Ireland, he was frequently consulted by letter, in cases of peculiar difficulty or danger.

Hardly any life, even of a literary man, can be conceived to afford fewer interesting materials for a biographer than Dr. Monro's. It was distinguished by no striking event, it was chequered by no vicissitudes of good and evil; it was a life, from early youth to extreme old age, of almost uniform and uninterrupted prosperity. Nay, he seems scarce to have felt any of those difficulties and discouragements in his splendid career, which most men of literary professions, but especially physicians, experience in their progress to the highest honours and rewards to which they can aspire; and certainly his progress never was retarded by any such adverse circumstances. His success, on all occasions, like the victories of Timoleon, seemed always to be accomplished with ease; yet it cannot, on this account, be attributed altogether to good fortune, or mere chance. Some favorable, almost accidental, circumstances contributed, no doubt, to his great success in life; but much more of it must be attributed to his own merits; to his constant unexampled activity in every pursuit in which he engaged; and to his good sense in perceiving and improving those advantages which might be considered as mere favours of fortune.

It must be useful and instructive, and, therefore, in some respects interesting, to know what fortunate circumstances, and what merits of his own, chiefly raised him to that eminence which he so long maintained. By his father, he was from his earliest youth carefully instructed in anatomy, and soon acquired such a taste for that science, and prosecuted the study of it with such ardour and perseverance, that, in the year 1753, he assisted his father in his anatomical lectures in the University; and, in the year 1755, was appointed conjunct professor of anatomy and surgery along with his father, who resigned the office entirely to him in 1758. From that time he continued to teach regularly for more than fifty years. His lectures were attended by vast numbers of students, generally from 200 to 400 every year. But, in the whole time, fifty years, or more, that he taught anatomy and surgery, his lectures were attended, in all, by fourteen thousand students.

This, of itself, may well be regarded, as a good proof of the merit and usefulness of his lectures, which indeed were conducted on a plan of the most extensive utility. It may well be doubted whether so useful a preliminary and preparation for the study and practice of physic and surgery ever had been given before in the form of lectures, or in any other form.

Dr. Monro's acknowledged merit as an anatomist, and as a teacher of surgery, though he was not himself a practical surgeon or operator, almost immediately brought him into very extensive practice as a consulting physician in the more important and difficult chirurgical cases; and in many cases, strictly speaking, not chirurgical, as not requiring or admitting of relief by any kind of manual operation; but for the complete understanding of which, with a view either to prognosis or practice, the most accurate anatomical knowledge is peculiarly requisite.

The quickness and clearness of his perception in such matters, his strong good sense, his decisive judgment, and the intimate knowledge of every part of the practice of physic, which he uniformly displayed on those occasions, soon, and most deservedly, procured him general confidence, and very extensive practice as a physician.

Perhaps no man of the medical profession ever more strongly illustrated by his conduct, and by the general tenor of his practice, the important truth, that the most valuable part of a physician's merit is good common sense steadily employed on a particular subject. If that one essential requisite be wanting, learning, science, genius, and every other accomplishment, are of no avail.

It is recorded of an ancient Greek physician, whose name (Trophilus) and whose apophthegm, but none of whose writings, have descended to us, that when he was asked 'Who would be a perfect physician?' he answered 'He who is able to distinguish what can be done, and what cannot be done.' As this apophthegm has been preserved, it is plain that his countrymen and contemporaries perceived the truth, acknowledged the merit, and felt the force of it: as every physician must do at once, who has been much engaged in practice, and who has fairly attended to what he saw.

But most physicians seem never to have known, or obstinately to have disregarded, that most important and almost self-evident truth; and very few of them have had the merit of regulating their practice according to that most precious maxim.

This merit Dr. Monro possessed in a very high degree. His just notions of general science, his thorough knowledge of medical science, but especially of anatomy, and of that part of pathology, with respect to the nature and causes, and seats of disease, which is ascertained by the dissection of morbid bodies, or what is often called morbid anatomy; and most chiefly his own strong good sense, and constant habit of strict attention and accurate observation, in his own practice, enabled him to perceive at once the futility of many hypothetical and erroneous, but very fashionable and prevailing doctrines, with respect to the nature and causes of many diseases; and of course led him to distrust, and often to reject, as unavailing at least, if not hurtful, many supposed remedies, and modes of practice, which were generally employed in consequence of those hypothetical dogmas.

The same accomplishments equally prompted him, and enabled him, to appreciate the real merits of many remedies which, from time to time, were introduced into practice with the most extravagant applause, purely on empirical principles; that is, on experience, real or supposed, of their good effects, without even an attempt to account for those supposed effects, or any pretence to explain their mode of operation.

Thus most effectually preserved from two of the worst and most frequent causes of bad medical practice, *his* had the merit of being simple, rational, and powerful; perhaps as powerful, and consequently as successful, as can be employed in the present imperfect state of medical science. But even the being spared the misery of enduring the administration of many unavailing remedies, bad if only frivolous, but much worse if severe also, was a matter of infinite consequence to his patients. Of his merit as a practical physician, when acting singly, his professional brethren, who have had occasion to consult with him, cannot fail to have a very high and just notion, when they recollect what his conduct was in consultation. Without any subtile disquisitions, without any controversies about obscure or disputed points, without any credulity as to the virtues of particular remedies, and far above the miserable vanity of arrogating to himself any superior skill, or pretending to extraordinary success in his own practice, he was generally the first to propose, and always was ready most candidly to agree with others who proposed, those simple and powerful modes of practice, from which alone, in urgent and dangerous cases, any essential benefit can be obtained. And in the many hopeless cases, in which his assistance was required, he was generally the first to observe that, 'little or nothing can be done in this case;' or, 'that there is no room for active practice here.'

Such remarks invariably led to the very humble and modest, but only rational practice which can be employed in these cases, the administration of remedies which may alleviate the sufferings of the patient, though they cannot cure the disease."—(*Memoir, pp.* ix.—xii.)

The second part of the work before us consists of the essays and heads of lectures of Dr. Monro, Secundus, enriched and illustrated as they are throughout by copious and valuable notes from the pen of the editor, Dr. Monro, Tertius. Of this portion of the work we feel that it would be difficult, if not impossible, to give a regular analysis. It embraces such a variety of important subjects, and comprises so much minute information, anatomical, physiological, pathological, and practical, that our limits would not do justice to it. We feel, therefore, that we are best discharging our duty to our readers by referring, and recommending them, to the work itself. In fact, as a standard book, and for occasional consultation, it ought to find a place in every medical library. If it should be objected by some that the opinions and labours of the late Dr. Monro belong to the last age, if not to the last century, and have been superseded by more captivating, and more modern, if not more accurate, researches; we would reply that the objection is met in limine by the real truth that the learned editor has everywhere added, in notes, such explanations of his father's doctrines, and (when needful) such corrections of them, as the progress of time, and the advancement of science, have rendered desirable. The work therefore, in point of fact, has quite a modern aspect; for the editor's notes, together with the communications of his various distinguished correspondents, bring up every thing, whether speculative or practical, *au courant du jour;* and thus superadd the attraction of novelty to the more solid and lasting merits of the volume.

On looking back, aided by all the advantages of our modern lights, at what the late Dr. Monro did and taught, no one can fail to perceive that he must have been a man of wonderful sagacity, and greatly in advance of his age. Long before the diagnostic discoveries of Laennec had dawned upon the world, Dr. Monro practised exploration of the chest, by manual examination, percussion, and agitation of the body. This circumstance is verified by the testimony of Sir Charles Bell and Dr. Alison of Edinburgh, both of whom had attended patients with him, labouring under diseases of the thorax. Moreover, he taught in his lectures, in the most distinct and emphatic matter, *the identity of human cow-pox and small-pox* ; in other words, that cow-pox is merely small-pox, modified by transmission through the cow ; a most important doctrine which has been verified by the experiments of Mr. Ceely (an eminent surgeon of Aylesbury*) only within the last two years. Such facts as these, in the estimation of the profession, and of all posterity, must stamp his fame.

* See Vol. VIII. of Transactions of the Provincial Medical Association.

